# Mechanical, Thermal, and Physicochemical Properties of Filaments of Poly (Lactic Acid), Polyhydroxyalkanoates and Their Blend for Additive Manufacturing

**DOI:** 10.3390/polym16081062

**Published:** 2024-04-11

**Authors:** L. Itzkuautli Mondragón-Herrera, R. F. Vargas-Coronado, H. Carrillo-Escalante, J. V. Cauich-Rodríguez, F. Hernández-Sánchez, C. Velasco-Santos, F. Avilés

**Affiliations:** 1 Centro de Investigación Científica de Yucatán, A. C., Materials Department, Calle 43 No. 130 x 32 y 34, Col. Chuburná de Hidalgo, Mérida 97205, Yucatán, Mexico; itzkuautli@hotmail.com (L.I.M.-H.); ross@cicy.mx (R.F.V.-C.); fhs@cicy.mx (F.H.-S.); 2 División de Estudios de Posgrado e Investigación, Tecnológico Nacional de México Campus Querétaro, Av. Tecnológico s/n, esq. Gral. Mariano Escobedo, Col. Centro Histórico, Santiago de Querétaro 76000, Querétaro, Mexico; cylaura@gmail.com

**Keywords:** poly (lactic acid), polyhydroxyalkanoates, polymeric filaments, additive manufacturing, mechanical, DMA

## Abstract

Polymeric blends are employed in the production of filaments for additive manufacturing to balance mechanical and processability properties. The mechanical and thermal properties of polymeric filaments made of poly (lactic acid) (PLA), polyhydroxyalkanoates (PHA), and its blend (PLA–PHA) are investigated herein and correlated to their measured structural and physicochemical properties. PLA exhibits the highest stiffness and tensile strength, but lower toughness. The mechanical properties of the PLA–PHA blend were similar to those of PLA, but with a significantly higher toughness. Despite the lower mechanical properties of neat PHA, incorporating a small amount (12 wt.%) of PHA into PLA significantly enhances toughness (approximately 50%) compared to pure PLA. The synergistic effect is attributed to the spherulitic morphology of blended PHA in PLA, promoting interactions between the amorphous regions of both polymers. Thermal stability is notably improved in the PLA–PHA blend, as determined by thermogravimetric analysis. The blend also exhibits lower cold crystallization and glass transition temperatures as compared to PLA, which is beneficial for additive manufacturing. Following additive manufacturing, X-ray photoelectron spectroscopic showed that the three filaments present an increase in C–C and C=O bonds associated with the loss of C–O bonds. The thermal process induces a slight increase in crystallinity in PHA due to chain reorganization. The study provides insights into the thermal and structural changes occurring during the melting process of additive manufacturing.

## 1. Introduction

Additive manufacturing is the process in which three-dimensional pieces are created by layering material based on a digital model [1,2,3,4,5,6,7,8]. One commonly used method for processing polymers by additive manufacturing is fused deposition modeling (FDM), where a filament of the material flows through a nozzle. Above the melting temperature (T_m_), the material is fused, deposited on a platform, and stacked layer by layer to form a three-dimensional object [2,3,4,5,6]. FDM has several advantages for polymer processing including the use of relatively simple equipment, a fast and low-cost process, high dimensional stability, good spatial resolution, and the ability to use filled or reinforced polymer filaments [3,4]. However, the limitations of FDM for polymers include presence of porosity, weak interfacial bonding, and a maximum operating temperature around 400 °C [3,5]. Polymers commonly used in FDM include acrylonitrile butadiene styrene (ABS), polylactic acid (PLA), polycarbonate (PC), polystyrene (PS), polypropylene (PP), polyethylene terephthalate (PET), polymethyl methacrylate (PMMA), nylon, and thermoplastic polyurethanes (TPU) [3,6]. The main drawback of using polymers in FDM for prototyping is their relatively low mechanical properties [7,8,9]. Therefore, polymer blends have been proposed to enhance and tailor the filament properties. PLA is the most commonly used polymer filament for FDM. For PLA, an interesting alternative is the use of polyhydroxyalkanoates (PHA) mixed with PLA [10,11].

**Figure 1:**
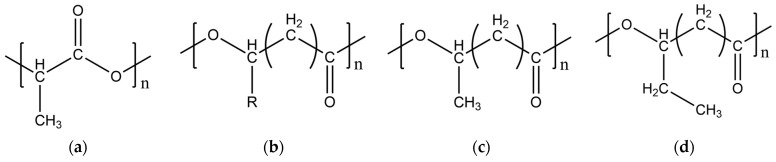
Chemical structure of the polymers. (**a**) PLA, (**b**) PHA, (**c**) PHB, (**d**) PHV.

PLA (Figure 1a) is a thermoplastic primarily made from cornstarch, sugarcane, or cellulose; it is considered biodegradable as it is derived mainly from biomass. Nevertheless, its suitability for load-bearing applications is restricted by its low toughness [12]. Additionally, its mechanical and rheological properties depend on its glass transition temperature (T_g_) [13]. On the other hand, PHAs (Figure 1b) are a family of hydroxyalkanoates. They are thermoplastic biopolymers synthesized by microorganisms through bacterial fermentation of lipids and sugars. The most studied PHA is polyhydroxybutyrate (PHB) (Figure 1c), which has a softening temperature (T_m_) between 173 and 180 °C and T_g_ between −51 and 15 °C [13], depending on its type and chain length. Another commonly studied PHA, often mixed with PHB, is polyhydroxyvalerate (PHV), providing greater flexibility to PHB. The most well-known PHA copolymer is poly(hydroxybutyrate-co-hydroxyvalerate) (PHBV). It has been reported that increasing the percentage of PHV in the PHBV copolymer significantly decreases T_m_ [14], although its crystallinity has minimal variation due to isodimorphism in the copolymer [15].

The PLA–PHA blend is a polymer mixture designed to achieve synergy in the properties of the blend, aiming for increased mechanical properties and high flexibility [14]. In general, the PLA–PHA blend is partially immiscible and its miscibility depends on the PHA concentration [14,16,17]. The morphology and microstructure of the blend depend on the relative proportions of each polymer. It has been reported that PHA contents less than 30 wt.% in the blend results in a nodular structure, while higher percentages of PHA create co-continuous structures [16]. Noda et al. [14] observed a significant increase in the toughness of the blend by adding 10 to 20 wt.% of PHB and polyhydroxyhexanoate copolymer to PLA. In addition to this, Gerard et al. [18] achieved similar mechanical properties using the same concentrations as employed by Noda et al. [14], attributing this to the formation of PHA spherulites of diameter less than 1 µm within the PLA. In blends with higher concentrations of PHA no particles were present, leading to significantly inferior properties. The mechanical and thermo-mechanical properties of parts manufactured by FDM can vary after undergoing the thermal melting process necessary for FDM [19]. Therefore, the study of the crystallinity and thermal properties of polymers and their relationship with the mechanical and dynamic mechanical properties of the filament is a topic of great interest that has been relatively underexplored. While there are numerous studies on the mechanical properties of standard coupons and FDM-printed parts, very few directly address the properties of the native filaments [20]. Therefore, this work focuses on analyzing the mechanical, dynamic mechanical, and thermal properties of PLA, PHA, and PLA–PHA polymeric filaments. Such material responses are explained herein based on their physicochemical properties measured using infrared and Raman spectroscopies, X-ray diffraction and X-ray photoelectron spectroscopy. The study also investigates the role that each component plays in the blend’s properties and the effect of thermal processing on the filaments.

## 2. Materials and Methods

### 2.1. Materials

The investigated polymers are commercial FDM filaments made of PLA (green color), PHA (black color), and a PLA–PHA blend (white color), all from ColorFABB (Belfeld, The Netherlands). As will be further discussed in the results section, the PHA filament is primarily composed of PHBV, according to the analyses conducted herein. However, the generic name of the polymer family (PHA) is maintained throughout this work to comply with its commercial nomenclature. The PLA–PHA filament is a blend with a mass ratio of 88:12 (PLA:PHA), with the PHA component predominantly composed of PHB [21].

### 2.2. Methods

#### 2.2.1. Physicochemical Analysis

The polymeric filaments were characterized using Fourier transform infrared spectroscopy (FTIR) in attenuated total reflectance mode. Spectra were obtained with a Nicolet Protégé 8700 spectrophotometer (Thermo Scientific, Waltham, MA, USA) and a zinc selenide crystal by averaging 100 scans in the 4000 to 650 cm^−1^ range with a resolution of 4 cm^−1^.

Raman characterization was performed with a Raman inVia confocal spectrometer (Renishaw, Sheffield, UK) in a range of 100 to 3200 cm^−1^ using a green laser; wavelength of 532 nm; 50× objective; exposure time of 10, 20, and 60 s for PHA, PLA, and PLA–PHA, respectively; and 1800 mm^−1^ grid. Three samples of each filament were analyzed using FTIR and Raman spectroscopies.

Wide-angle X-ray diffraction (WAXD) analysis was conducted in a D-8 Advance diffractometer (Bruker, Billerica, MA, USA) using a diffraction angle (2θ) range of 5° to 50°, CuK α radiation (λ = 0.154 nm) at 40 kV, 30 mA, a step size of 0.02°, and a step time 0.05 s. For each material, six samples were analyzed; three of them were obtained from the cross-section of the filament (hereafter referred to as “FIL”), and the other three extracted from a square of 10 mm side-length and 0.4 mm thickness manufactured using fused deposition modeling (hereafter referred to as “FDM”). The FDM specimens were manufactured with a nozzle diameter of 0.4 mm, layer height of 0.16 mm, extrusion temperature of 200 °C, heat bed temperature of 60 °C, and extrusion speed of 20–25 mm/s. The specimens comprise two polymer layers with printing directions of 45° and −45°.

The WAXD crystallinity was calculated from the areas of the crystalline and amorphous peaks [22] as follows:

(1) $$X_{c}^{WAXD}=\frac{A_{c}}{A_{c}+A_{a}}$$

where *A_c_* is the sum of the areas of all crystalline peaks, and *A_a_* is the sum of the areas of all amorphous halos.

X-ray photoelectron spectroscopy (XPS) was conducted using a equipment model K-ALPHA (Thermo Scientific, Waltham, MA, USA) with an argon ion gun at a voltage of 3 kV and an aperture of 400 nm. Surface erosion was carried out with an argon beam for 15 s accelerated to 3 kV and a power of 30 W over an area of 1 mm × 2 mm. The first analysis was a general survey with scanning from 0 to 1350 eV with a step size of 1 eV and an energy step of 100 eV. Subsequently, specific detailed scans (windows) were analyzed for carbon (282–291 eV) and oxygen (529–536 eV), with a step size of 0.2 eV and an energy step of 50 eV. Deconvolutions were performed using Lorentz functions. For XPS, three filament samples (FIL) and three additive manufacturing samples (FDM) were analyzed for each material.

#### 2.2.2. Thermal Analysis

Differential scanning calorimetry (DSC) was carried out in a DSC 7 calorimeter (Perkin Elmer, Shelton, CT, USA). Samples were analyzed inside sealed aluminum pans from 40 °C to 200 °C with a heating rate of 10 °C/min under a nitrogen atmosphere. To investigate the effects of thermal history, two consecutive heating programs were performed for each analyzed sample. The DSC crystallinity ($X_{c}^{DSC}$) was obtained from the enthalpy measurements as described in [23]:

(2) $$X_{c}^{DSC}=\frac{{∆H}_{f}-{∆H}_{cc}}{{∆H}_{f}^{0}}$$

where $∆H_{cc}$ is the enthalpy of cold crystallization, $∆H_{f}$ is the calculated fusion enthalpy (obtained from the area under the melting peak), and ${∆H}_{f}^{0}$ is the fusion heat for a 100% crystalline polymer. $H_{f}^{0}$ was considered as 93.7 J/g for PLA [23] and 109 J/g for PHBV [24]. For PHA $∆H_{cc}$ = 0 because this polymer does not undergo cold crystallization. According to Nanda et al. [11], the miscibility of two polymers comprising a blend can be estimated based on their *T_g_* and a miscibility parameter (*k*) as follows:

(3) $$T_{g}=\frac{W_{PLA}T_{gPLA}+kW_{PHA}T_{gPHA}}{W_{PLA}+kW_{PHA}}$$

where *T_gPLA_* and *T_gPHA_* are the glass transition temperatures of PLA and PHA, respectively; *W_PLA_* and *W_PHA_* are the corresponding weight fractions (wt.%) of PLA and PHA in the blend, respectively; and *T_g_* is the glass transition temperature of PLA in the blend. The parameter *k* is a fitting parameter that can be interpreted in terms of the miscibility of the system. According to Nanda et al. [11], *k* = 1 indicates high miscibility of the polymers in the blend, while a very high (well above 1) or very low value of *k* indicates low miscibility.

Thermogravimetric analysis (TGA) was conducted using a TGA 8000 instrument (Perkin Elmer, Shelton, CT, USA). For this analysis, 10 mg of each filament was heated in the range of 50 °C to 500 °C with a heating rate of 10 °C/min under a nitrogen atmosphere. Three replicates of each filament were analyzed.

#### 2.2.3. Mechanical and Dynamic Mechanical Characterization

The tensile properties of the polymeric filaments were measured using a AG-I universal testing machine (Shimadzu, Kyoto, Japan) with a 500 N load cell in a uniaxial tensile test at a crosshead speed of 20 mm/min. Special grips for cords were used, as Supplementary Figure S1 demonstrates. The mechanical analysis was conducted following the ASTM standard D3379 [25], with ten replicas per group. Initially, three groups of samples with different calibrated lengths (*L_g_*) were tested, namely, *L_g_* = 60, 120, and 180 mm. However, since they produced very similar results, only *L_g_* = 120 mm is reported herein. The calculation of the elastic modulus was determined from a linear fit of the stress–strain curve between 1% and 3% strain for all three types of samples.

The rule of mixtures was employed to further investigate compatibility/miscibility effects and determine the mechanical properties (elastic modulus, tensile strength, elongation at break, toughness) of the PLA–PHA blend as noted in [26]:

(4) $$\xi_{PLA/PHA}=v_{PLA}\xi_{PLA}+v_{PHA}\xi_{PHA}$$

where $\xi_{PLA/PHA}$ is the mechanical property of the blend; $v_{PLA}$ and $v_{PLA}$ are the volumetric fractions of PLA and PHA in the blend, respectively; and $\xi_{PLA}$ and $\xi_{PHA}$ are the mechanical properties of PLA and PHA, respectively.

Scanning electron microscopy (SEM) was employed to examine the fracture surface of the filaments. This study was conducted using a JSM-6360LV SEM instrument (JEOL, Tokyo, Japan) with an accelerated voltage of 20 kV. Specimens were observed under vacuum with a gold thin coating (~20 nm) for enhanced conductivity.

Dynamic mechanical analysis (DMA) was performed using a Q800 dynamic mechanical analyzer (TA Instruments, New Castle, DE, USA). The filaments were subjected to uniaxial tensile loading with a frequency of 1 Hz, a static load of 0.01 N, a dynamic load of 0.4 N, and a heating ramp of 3 °C/min from 30 °C to 120 °C (or up to 170 °C in the case of PHA).

## 3. Results and Discussion

### 3.1. Chemical and Structural Composition of the Filaments

#### 3.1.1. Fourier Transform Infrared Spectroscopy

**Figure 2:**
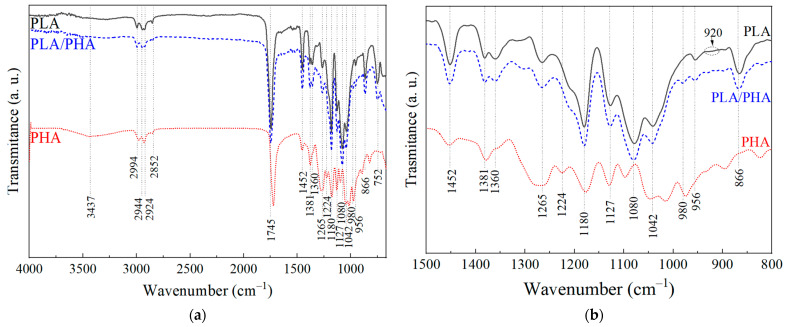
FTIR spectrum of the polymeric filaments (band locations are referenced to PLA–PHA). (**a**) 4000 to 600 cm^−1^ wide spectrum, (**b**) 1500 to 800 cm^−1^ window.

The FTIR spectra of the polymeric filaments are shown in Figure 2. The spectra were normalized with respect to the band at 1745 cm^−1^, corresponding to the carbonyl group present in the main chain of both polymers. The band at 3437 cm^−1^ (only present in PHA) is associated with O–H bond stretching [27,28]. Bands between 2994 cm^−1^ and 2852 cm^−1^ correspond to symmetric and asymmetric stretching of C–H in CH_3_ [27,28,29,30,31]; bands at 1721–1745 cm^−1^ relate to carbonyl group stretching [27,28,29,30,31,32,33]; bands between 1454 cm^−1^ and 1358 cm^−1^ are characteristic of symmetric and asymmetric deformations of the CH_3_ bond [27,28,29,30,33]; bands between 1269 cm^−1^ and 1079 cm^−1^ relate to deformations of the ether group, C-O-C [27,28,29,31,33]; bands from 955 cm^−1^ to 1041 cm^−1^ correspond to rocking of the C–CH_3_ bond [27,28,29,33]; bands at 895–865 cm^−1^ refer to stretching of the O-CH-CH_3_ group [27,28,29,30,33], and bands at 752–751 cm^−1^ correspond to wagging of CH_3_ (present in PLA and PLA–PHA) [27,30]. A more detailed summary of the band locations for each polymeric filament and its bonding/vibration type can be found in Table S1 of the Supplementary Information. Figure 2b provides a more detailed view of the 1500 cm^−1^ to 800 cm^−1^ region.

In the spectrum of PLA, bands at 956 cm^−1^ and 1180 cm^−1^ are indicative of its amorphism [33], while bands at 920 cm^−1^ and 1210 cm^−1^ are related to its crystallinity [34]. This suggests that PLA exhibits low crystallinity. In contrast, for PHA, bands at 980, 1224, 1275, and 1721 cm^−1^ indicate higher crystallinity [28,35,36], while the band at 1180 cm^−1^ is associated with amorphism [35]. This suggests that PHA is semi-crystalline. Moreover, the difference in intensity between the bands at 1723 cm^−1^ and 1745 cm^−1^ suggests that the PHA filament is composed of PHBV (79% PHB, 21% PHV, wt.%) [37]. Finally, the PLA–PHA spectrum bears greater resemblance to PLA than to PHA, consistent with its much higher mass concentration (88:12). The PLA–PHA spectrum exhibits bands from both polymers and is very similar to that of PLA. Most of the bands of PLA and PHA overlap, with the only appreciable difference present in the blend but not in PLA found in the band at 980 cm^−1^, related to the crystallinity of PHA.

#### 3.1.2. Raman Spectroscopy

**Figure 3:**
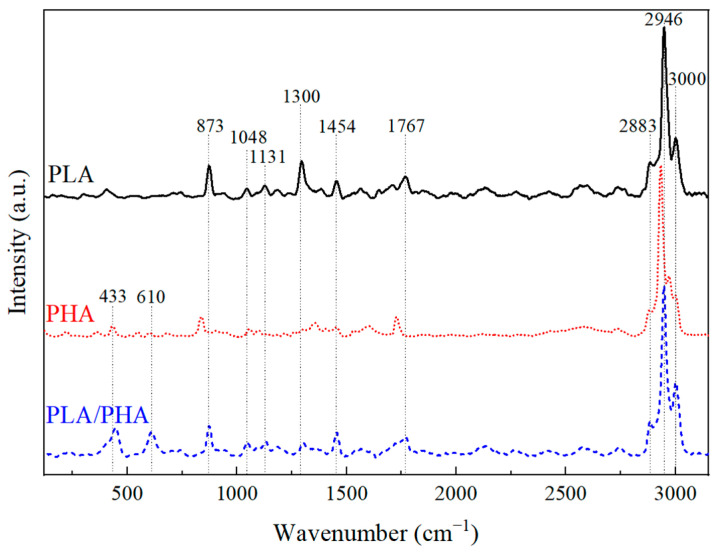
Raman spectra of the polymeric filaments (band locations are referenced to PLA–PHA).

Figure 3 shows the Raman spectra of the polymeric filaments. The spectra were normalized with the band at 2946 cm^−1^, which is related to CH_2_, corresponding to the main chain of both polymers. The Raman spectrum of PLA shows bands at 3001, 2946, and 2882 cm^−1^, corresponding to the stretching of CH, asymmetric stretching of CH_3_, and symmetric stretching of CH_3_, respectively [27,28]. Bands at 1767, 1455, 1298, and 874 cm^−1^ represent stretching of C=O, asymmetric deformation of CH_3_, symmetric deformation of CH_2_, and stretching of the C–COO bond, respectively [27,28].

PHA exhibits bands at 2998 cm^−1^ corresponding to the asymmetric stretching of CH_3_, 2993 cm^−1^ to the symmetric stretching of CH_2_, 2877 cm^−1^ to stretching of CH, 1730 cm^−1^ to stretching of the carboxylic group, 1455 cm^−1^ to the asymmetric deformation of CH_3_, 1361 cm^−1^ to symmetric deformation of CH_2_, 1100 cm^−1^ to in-plane bending of CH_3_ and symmetric stretching of C–O–C, 1055 cm^−1^ to symmetric stretching of the C–CH_3_ bond, 835 cm^−1^ to stretching of the C–COO bond, 610 cm^−1^ to the deformation of the C–CO and C–CH_3_ bonds, and 433 cm^−1^ to the skeletal deformation of the C–C bond [28,29,38].

On the other hand, the Raman spectra of the PLA–PHA blend exhibits the main features and bands of PLA with slight shifts in peak positions. Additionally, the blend shows bands at 1131, 1048, 613, and 448 cm^−1^, corresponding to in-plane bending of CH_3_, stretching of the C–CH_3_ bond, deformation of the C–CO and C–CH_3_ bonds, and skeletal deformation of the C–C bonds, respectively.

A detailed summary of the Raman vibrational bands for the three polymeric filaments is included in Table S2.

In the PLA spectrum, the width of the 1767 cm^−1^ band and its lower intensity compared to that at 874 cm^−1^ is related to a high amorphous content. In the case of PHA, this variation is observed in the 1730 cm^−1^ band relative to the 843 cm^−1^ band. Semi-crystalline materials have similar intensities between those two bands and are narrower, which is not the case herein. Furthermore, based on the relative intensities of the bands at 1361 and 1730 cm^−1^ in PHA, it can be inferred that the PHA filament is composed of PHBV, with an approximate weight ratio of 75–79% (PHB) and 25–21% (PHV) [28]. This ratio is consistent with the one calculated from the FTIR analysis.

#### 3.1.3. X-ray Photoelectron Spectroscopy

**Figure 4:**
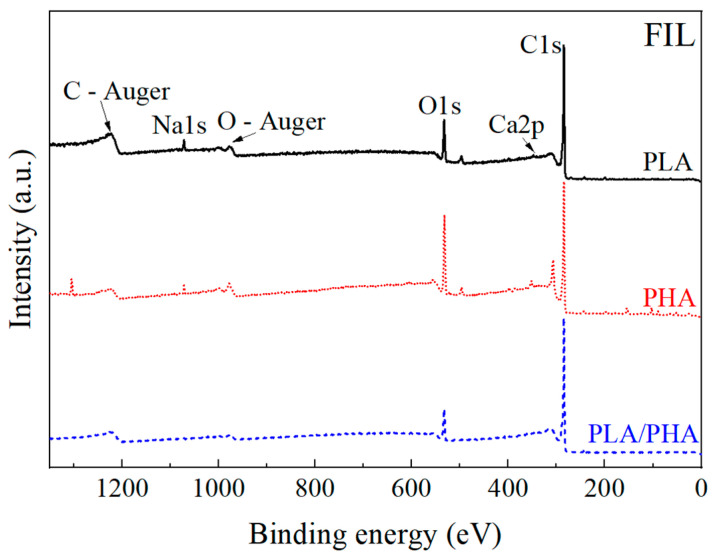
XPS survey spectrum of the polymeric filaments.

Figure 4 shows the results of the survey analysis of X-ray photoelectron spectroscopy (XPS) of the polymeric filaments. Two main bands of interest (carbon and oxygen) are observed for each sample. The survey spectrum shows the main carbon (~285 eV) and oxygen (~533 eV) bands, along with traces of some minor elements present in the samples, such as sodium (~1072 eV) and calcium (~347 eV), potentially originating from additives and/or colorants in the filaments. In the case of PLA, the variation between carbon and oxygen concentrations is higher than those reported in the literature [39]. This difference may be attributed to an excess of C–C bonds, suggesting the presence of pigments and/or additives (PLA is of green color) [40].

A detailed window analysis of C and O along with their deconvolutions is shown in Figure S2. In the case of carbon deconvolution, three types of bonds were identified, viz., C–C, C–O, and C=O. For the oxygen deconvolution, C–O and C=O bonds were present. A table with the concentrations of the main elements and bonds will be discussed in Section 3.2.4, where a comparison is made with samples manufactured using FDM.

#### 3.1.4. Wide-Angle X-ray Diffraction

**Figure 5:**
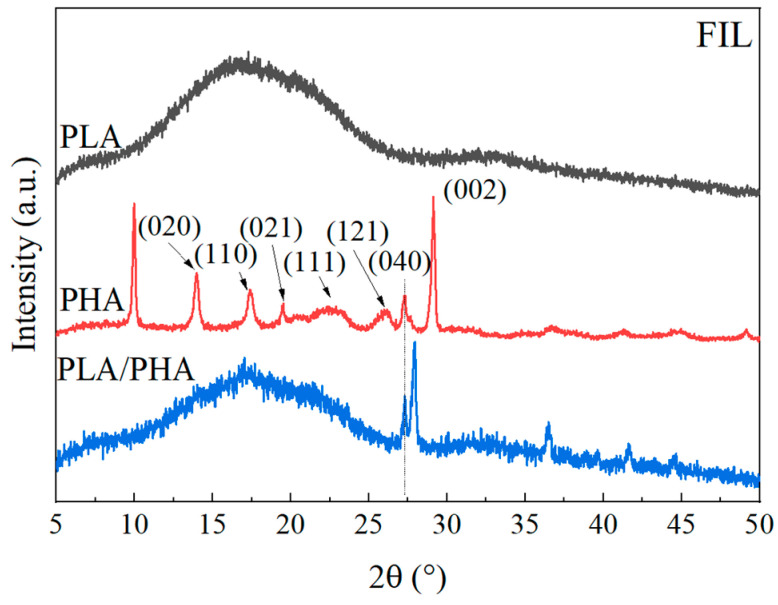
WAXD diffractograms of the polymeric filaments.

The WAXD diffractograms of the polymeric filaments are shown in Figure 5. In the PLA diffractogram, a broad halo is observed in the 10° to 25° 2θ range, suggesting the dominance of the amorphous part in this semi-crystalline polymer [41]. The center of this halo at 15.5° is related to the (200/110) plane [41,42], attributable to the α structure of PLA [42]. On the other hand, the PHA diffractograms exhibit characteristic crystalline peaks of the planes (020) at 2θ = 13.6°, (110) at 17.1°, (021) at 19.2°, (101) at 21.5°, (111) at 22.4°, (121) at 25.7°, (040) at 27.0°, and (002) at 30.7° [43]. The peaks at 2θ = 13.6° and 17.1° indicate orthorhombic unit cells [43,44]. The peak at 2θ = 22.4° corresponds to the α–PHB crystal, while 2θ = 25.7° and 27.0° correspond to the partially crystalline nature of PHB.

The PLA–PHA blend predominantly shows the amorphous halo of PLA, superimposed with the crystalline peaks of PHA from the orthorhombic cell (2θ = 13.6° and 17.0°) and its partially crystalline nature (2θ = 27.5°). The crystalline peaks (101) and (111) of the PHA correspond to PHBV, with a possible ratio of 79:21 [44], as observed in both FTIR and Raman. Equation (1) yields a crystallinity of 45 ± 4% for PHA, which is related to the PHB portion.

### 3.2. Thermal Properties and Effect of Melting by Additive Manufacturing

#### 3.2.1. Differential Scanning Calorimetry of Filaments

**Figure 6:**
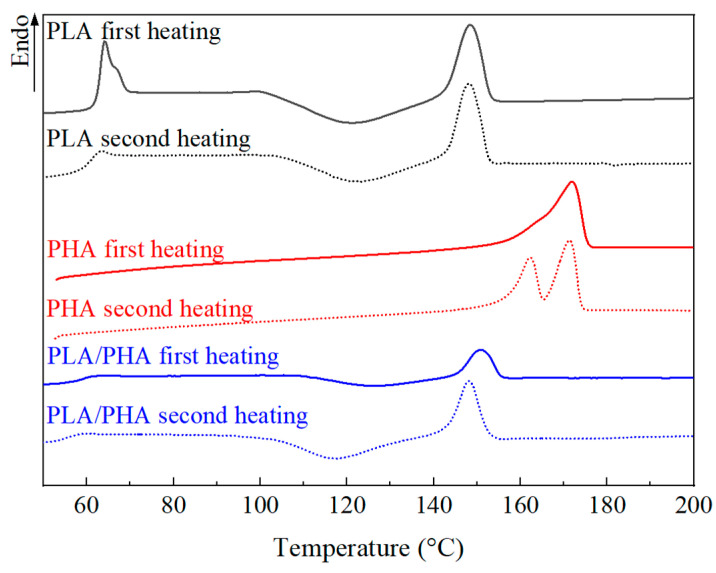
DSC thermograms of the polymeric filaments.

Figure 6 shows the DSC thermograms of PLA and PHA for the first and second heating cycles. Additionally, Table 1 presents the average values obtained for the glass transition temperature of PLA (*T_g_*), cold crystallization temperature of PLA (*T_cc_*), and the melting temperatures of PLA (*T*_*m*1_) and PHA (*T*_*m*2_, *T*_*m*3_). In the PLA thermograms, the differences between the first and second heating are minimal, suggesting very little or no variation in the polymer’s crystallinity after a thermal process reaching the melting temperature. This melting process could emulate what happens in FDM. For PHA, specifically PHBV, T_g_ is not observed since it falls between −10 and 5 °C [16,45], and the analysis started at 50 °C. In the first heating of PHA, the T_m_ of the orthorhombic crystalline structure [22,46] is seen along with a shoulder, which is related to the β crystalline structure [22]. In the second heating, the presence of both T_m_ peaks is related to two PHBV polymorphs, PHB and PHV. The two endothermic peaks for T_m_ correspond to the orthorhombic (PHB) and β (PHV) crystalline structures, as observed in XRD. PHB has a low nucleation level, which tends to form small spherulites with different structure than PHV [47]. In the DSC thermograms of the PLA–PHA blend, a slight shift towards lower temperatures is observed compared to PLA, with shifts of 3 °C and 5 °C in T_g_ and T_cc_, respectively. These shifts may be related to an increase in the mobility of the PLA chains due to interaction with the PHA chains, attributed to the miscibility of the amorphous phases. The T_m_ shown in the respective blend of PLA remains the same, and that of PHA is not prominent, possibly due to the low concentration of PHA present.

Consistently with WAXD, DSC indicates that the crystallinity of PLA is very low. On the other hand, the crystallinity of PHA shows similar values in both techniques, i.e., ~46% for WAXD and ~48% according to DSC. The increase in crystallinity in the second heating cycle in PHA may be related to the degradation, which enhances chain mobility and facilitates crystallization during cooling between heating cycles [48].

Equation (3) was used to estimate the miscibility of the PLA–PHA blend using the measured *T_g_* of PLA (63.5 °C), the measured *T_g_* of PLA in the blend (60.5 °C), the corresponding weight fractions of the blend (88% PLA, 12% PHA), and *T_g_* of PHA taken from the literature. According to the analyses shown herein, the PHA filament used is mostly PHBV with a relation 79:21 (PHB:PHV). Hence *T_gPHA_* = −1 °C was considered, as reported in [49] for a PHB:PHV ratio of 80:20. With this input, Equation (3) yields *k* = 0.36. It is important to note that, even if a broader range for *T_g_* of PHBV is considered, such as −10 to 5 °C as reported in [16,45], *k* is bounded between 0.31 and 0.39. This result further supports the partial miscibility of the blend since *k* = 1 represents total miscibility [11]. Such a partial miscibility is expected between the amorphous regions of both polymers, rather than within their crystalline zones.

#### 3.2.2. Thermogravimetric Analysis of Filaments

**Figure 7:**
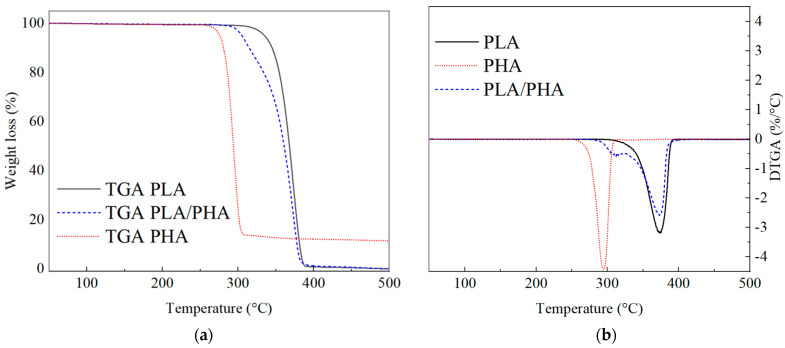
TGA thermograms of the polymeric filaments. (**a**) Mass loss as a function of temperature; (**b**) Derivative of mass loss as a function of temperature.

Figure 7 shows the TGA thermograms of the polymeric filaments. Table 2 lists the initial degradation temperatures of PLA (T_id1_) and PHA (T_id2_), the maximum degradation temperatures of PLA (T_dmax1_) and PHA (T_dmax2_), and the final degradation temperatures of PLA (T_fd1_) and PHA (T_fd2_), as well as the remaining (residual) material at 500 °C. In the case of PLA, T_id1_ = 319.3 °C, T_dmax1_ = 373.7 °C, and T_fd1_ = 390.3 °C. For PHA, T_id2_ = 262.0, T_dmax2_ = 296.3, and T_fd2_ = 315.7. Additionally, the PHA sample after T_fd2_ retains ~12% of mass, likely from chain scissions of C=O and C–O bonds in ether fractions forming short chains of carboxylic acids with olefinic end groups, oligomers, and crotonic acid [31,50]. In the PLA–PHA blend, the first degradation temperature is observed at T_id1_ = 292.0 °C, with a corresponding T_dmax1_ = 315.0 °C. This first degradation (292–330 °C) corresponds to the PHA portion, while the second degradation (331–395 °C) is related to the PLA portion. The second degradation occurs with T_id2_ = 331.0 and T_dmax2_ = 374.0 °C. T_fd_ of PLA–PHA is 394.7 °C. From this, it is inferred that the onset of PHA degradation occurs approximately 57 °C before PLA. However, when mixed with PLA this difference decreases, providing thermal stability. On the other hand, after T_fd_ in PLA and PLA–PHA, only ~1% of mass remains, corresponding to carbonaceous residues, impurities, and filament additives.

Regarding the results of the derivative of mass loss as a function of temperature (DTGA, Figure 7b), a synergistic effect is observed in PLA–PHA because it exhibits the highest thermal stability, followed by PLA and PHA. The values are approximately ~26%/°C (at ~374 °C), ~32%/°C (at ~374 °C), and ~44%/°C (at ~297 °C), respectively.

#### 3.2.3. X-ray Diffraction of Polymers after FDM

**Figure 8:**
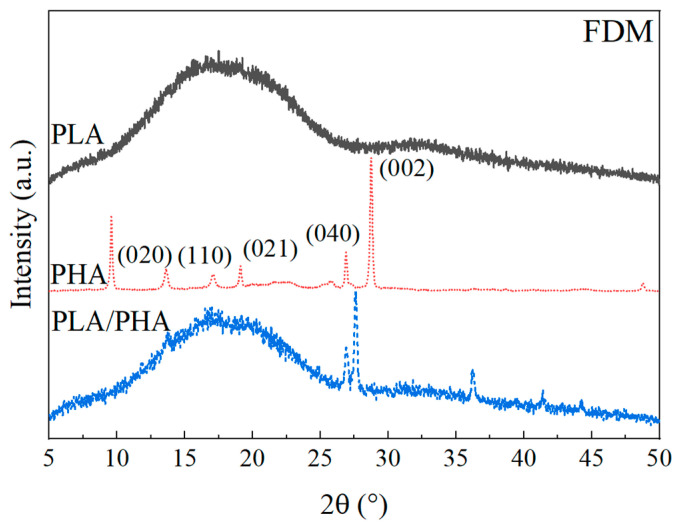
WAXD diffractograms of the FDM samples.

The WAXD diffractograms of the samples processed by FDM are shown in Figure 8. In the PLA diffractogram, an amorphous halo similar to that of the filament before undergoing the FDM process is evident (Figure 5). However, it exhibits a ~2° shift to the right, suggesting a small rearrangement of the PLA chains during the FDM process. In the case of PHA, there is an increase in the relative intensity of the peaks at 9° and 28°, compared to the diffractogram of the PHA filament (Figure 5). These peaks are related to the crystallinity of PHB, indicating chain alignment and growth of PHA crystallinity, which may be affected by the extrusion and printing direction [19].

This increase in crystallinity was also observed by DSC (Table 1). Therefore, it is suggested that the peaks in PHA that decreased or disappeared (13°, 17°, 19°, 22°, and 25°) are associated with the amorphous part of PHBV. In the case of the blend, the amorphous halo related to PLA remains unchanged, and the broad peak in the orthorhombic structure is maintained. For the PHA part of the blend, the peak related to the crystalline nature of PHB is retained. The peaks of the orthorhombic cell of PHA (13° to 25°) are not observed as they are in the same region as the amorphous halo of PLA, and their intensity has decreased, as observed in pure PHA. Using Equation (1), the crystallinity of PHA is calculated as 52 ± 4%, showing an increase of ~5% with respect to the filament discussed in Section 3.1.4. This increase may be related to chain alignment during the FDM process, consistent with the observed increase in crystallinity in DSC (Table 1).

#### 3.2.4. X-ray Photoelectron Spectroscopy

**Figure 9:**
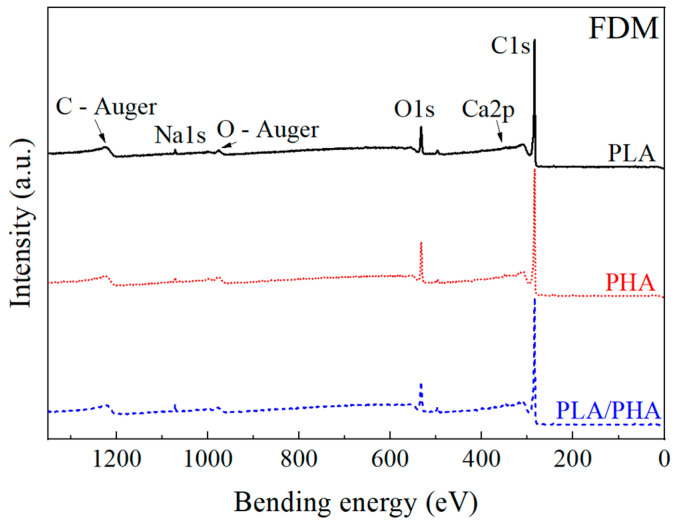
General XPS spectrum of the samples using FDM.

Figure 9 shows the results of the XPS survey analysis of the FDM samples. Similar to the XPS spectra of pristine polymeric filaments shown in Figure 3, the main bands are carbon and oxygen.

Table 3 presents the elemental concentration and the atomic C/O ratio of pristine polymeric filaments (FIL) and samples produced using FDM. The values shown in this table have a coefficient of variation that averages 9%. It is observed that PHA has the lowest C/O ratio, indicating a higher presence of oxygenated groups. This is supported by the 3437 cm^−1^ FTIR band corresponding to O-H stretching vibrations, which is seen only in PHA. The pristine PLA filament has an average C/O ratio of 7.15, which increases to 9.76 when processed using FDM. In the literature, the C/O ratio of PLA has been reported with values close to 2 [51,52]. The higher value of the investigated commercial filament in this study may be attributed to the green dye in the filament. On the other hand, additional elements present in the samples, such as calcium, sodium, chlorine, and nitrogen, may indicate filament additives, such as green (PLA), black (PHA), and/or white (PLA–PHA) dyes. Due to the high surface sensitivity of XPS, small traces (magnesium, chlorine, silicon) from the tools used for obtaining samples and/or the metallic printing nozzle cannot be ruled out. In all three polymeric matrices, FDM samples exhibit a higher C/O ratio, corresponding to the loss of oxygenated groups during the thermal process of additive manufacturing.

Table 4 presents the relative bond concentration found in each of the samples analyzed by XPS. For the C1s band, three types of contributions were found, viz., C–C (~285 eV) [51,52], C–O (~286 eV) [51,52], and C=O (~289 eV) [51,52]. The carbon-carbon (C–C) and carbon-hydrogen (C–H) bond represents methylene (CH_2_), methyl (CH_3_), and the bond between carbons in the main chain, as well as those of the pendant groups. The carbon-oxygen bond (C–O) is related to the ether bond (C–O–C) in the main chain and part of the carboxylic group (–COOH). Meanwhile, the carbon-oxygen double bond (C=O) indicates the presence of the carbonyl group in the ester. These three bonds appear in all three polymeric matrices. In the case of the O1s band, the contribution of two bonds is observed, namely, C–O (~533 eV) [51,52] and C=O (~534 eV) [51,52].

For the C1s band of FDM-processed PLA and PLA–PHA, an increase in C–C groups and a decrease in C=O and C–O bonds are observed when compared to their corresponding pristine filaments. Similarly, after undergoing the FDM process, a greater decrease in the carbonyl bond in the O1s band is observed. Both effects are associated with the breaking of ester bonds and chain scission. On the other hand, in the C1s and O1s bands of PHA, nearly constant values of C–C bonds and an increase in the C=O bonds are observed. This finding is noted because, when subjected to a thermal process, PHA tends to form short chains made of carbonyl and carbon–carbon bonds [50,53]. The formation of chains is related to the behavior of PHA at high temperatures and attributed to the formation of carbonyl group chains. This contrasts with the total degradation observed in PLA, a phenomenon noted in the thermogravimetric analysis (Figure 7).

### 3.3. Quasi-Static Tensile Response of Filaments up to Failure

**Figure 10:**
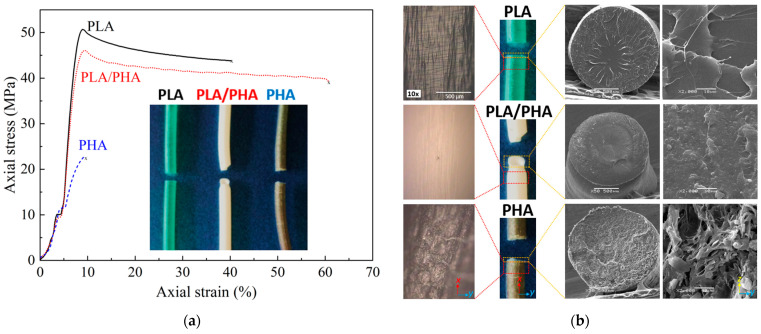
Response to axial loading of polymeric filaments. (**a**) Stress–strain curve, (**b**) optical and SEM microscopy of the fractured filaments.

Figure 10a shows the stress-strain curve of the axial tensile response of the polymeric filaments. A summary of the results obtained is listed in Table 5. PLA exhibits the highest tensile strength and stiffness, with average values of 50.6 MPa and 1.5 GPa, respectively. As seen from the inset and fracture images of Figure 10b, the filament failed with little necking and at angle almost perpendicular to the loading direction, suggesting that the ductility of the PLA filament is rather limited. Microcracks bridged by polymer fibrils (“crazes”) are seen for PLA in Figure 10b, indicating that the material is not completely brittle but has certain ductility. On the other hand, PHA shows the lowest mechanical properties and a brittle fracture surface, with an average tensile strength and elastic modulus of 21.1 MPa and 0.5 GPa, respectively. Micrographs of PHA (Figure 10b) reveal the formation of microcracks and brittle failure. The SEM micrographs show the presence of micropores, which may be related to the mixture of PHB–PHBV and partial immiscibility between the crystalline and amorphous regions [53]. The poor mechanical properties and brittle response of PHA yielded failure close to the looping grips of the filament test rig for most of the specimens (see Figure S1). Regarding this, Rodrigues et al. [20] stated that to prevent fracture in the winding section of the filament test rig, the elongation at break must be greater than the ratio between the filament diameter and the fixture radius. In our case the filament diameter is 1.75 mm and fixture radius is 7.5 mm, which yields a minimum elongation at break of at least 23%. This value is higher than that of PHA (16.2%), supporting the experimental observations.

As seen from the PLA–PHA response in Figure 10a, adding a small amount of PHA (12 wt.%) to PLA greatly increases the elongation at break and toughness of the blend, albeit PHA is brittle. The average toughness is 16.1 J/m^3^ for PLA, 1.1 J/m^3^ for PHA, and 23.8 J/m^3^ for the blend. This is because PLA–PHA exhibits the highest elongation at break, exceeding 50%, concomitant with significant necking and ductile fracture at an inclined angle with respect to the loading direction (see Figure 10). The ultimate strain of PLA is higher than that presented in some literature reports using other manufacturing processes and/or specimen geometries, such as film extrusion with a value of ~3% [54], extruded filament at ~5% [55], and an injection-molded specimen with a value of ~2% [56], but similar to that of other commercial filaments [57,58]. This could be due to processing effects of the commercial filaments, proprietary additives and processing aids, and different test rigs and specimen geometry. The high toughness of PLA–PHA is a synergistic effect attributed to the distribution of PLA and PHA polymers in the blend with an approximate weight ratio of 88:12 (PLA:PHA), likely bonded by hydrogen bonds. Upon stretching the filament, these bonds also stretch and transfer mechanical load. Breaking these bonds and fracturing the PLA–PHA filament requires cavitation (decohesion of PHA spherulites) and/or crack deflection around the PHA phases, which are both recognized toughening mechanisms [59]. It has been reported that at this relative concentration, the PLA–PHA blend consists of PHA spherulites within the PLA, yielding mechanical compatibility between the polymers [18]. This spherulite formation was confirmed in our case through SEM microscopy (Figure 10b). Furthermore, the C=O groups of the PLA and the O-H groups of the PHA are prone to form CO–O bonding.

Zhao et al. [23] report that at concentrations between 15 and 30% of PHA within PLA, a single morphological phase is formed, allowing greater elongation before fracture compared to other mixing ratios. For higher concentrations of PHA, it is expected that the mixture will exhibit two phases in a co-continuous morphology with interpenetrated structures of each polymer [18,23]. The rule of mixtures, Equation (4), was also used to estimate the mechanical properties of the blend. Taking the density of PLA as 1.3 g/cm^3^ and PHA as 1.24 g/cm^3^, [60], the volume ratio of the blend is 87.5:12.5 (PLA:PHA), which was used in Equation (4). As observed from the last column of Table 5, the elastic modulus and tensile strength of the PLA–PHA blend is quite well predicted by the rule of mixtures. Discrepancies with average values are less than 7%. This agreement suggests partial miscibility between both phases of the blend. On the other hand, both the elongation at break and toughness measured for PLA–PHA are higher than those predicted by the rule of mixtures. This indicates the existence of synergy in the PHA–PLA blend, especially between the amorphous parts of both polymers.

### 3.4. Dynamic Mechanical Analysis of Filaments

**Figure 11:**
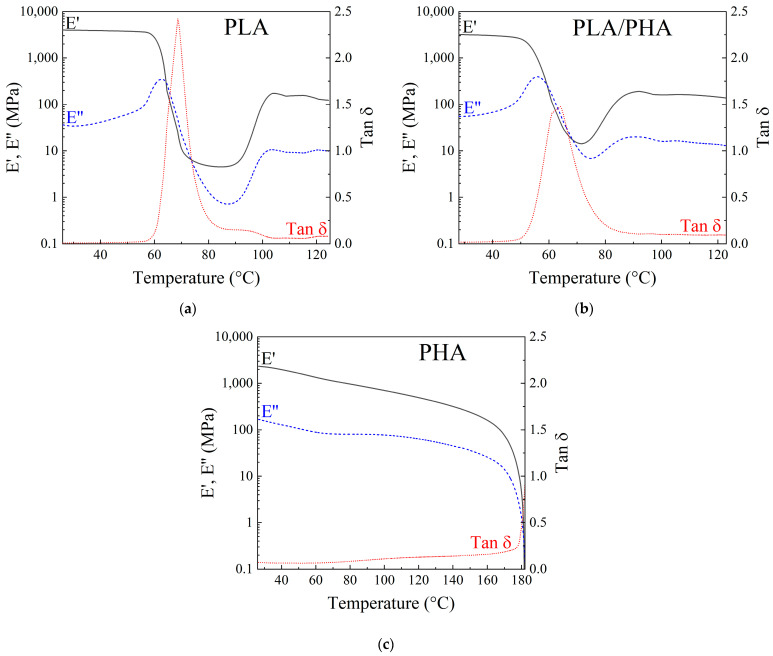
DMA of the polymeric filaments. (**a**) PLA, (**b**) PLA–PHA, (**c**) PHA.

Figure 11 shows the DMA results of the polymeric filaments, where the storage modulus (E’), loss modulus (E’’), and the damping factor (tan δ) are plotted as a function of temperature. E’ of PLA starts at 3.9 GPa at 25 °C, and it remains almost constant up to 60 °C. As the temperature increases from 60 to 68 °C, E’ sharply decreases by two orders of magnitude. This is related to the glass–rubbery transition [61], relative to the α relaxation of the amorphous regions of PLA [62]. From 68 to 90 °C, E’ of PLA only shows a slight decrease, known as the rubbery plateau [61]. Subsequently, from 90 to 100 °C, E’ of PLA increases ~30 times. This increase is related to the recrystallization transition, characteristic of PLA [61]. Finally, for temperatures above 100 °C, E’ remains almost constant, a stage that has been referred to as the “rubber state with crystals” [61]. This is the temperature where PLA crystallization occurs, as observed in the DSC. In the case of E’’ for PLA, it starts with ~35 MPa at 25 °C; at 60 °C, it increases ~25 times, which is attributed to the glass transition temperature. Following this, it decreases by two orders of magnitude starting from 63 °C and increases again at 86 °C. Similar to E’, these stages are known as the glass–rubbery and recrystallization transitions, respectively. In tan δ, a peak centered at 68 °C with a large area under the curve is observed, indicating α relaxation due to the high chain mobility [61,63]. The glass transition temperature (T_g_) estimated from the peak in tan δ yields 68 °C, which compares well with that determined using DSC (64 °C).

On the other hand, the onset of the rubber state with crystals is related to the onset of cold crystallization (T_cc_), which in the DMA corresponds to 101 °C. This again matches that determined by DSC (T_cc_ = 101 °C). For the PLA–PHA blend (Figure 11b), the DMA response is quite similar to that of PLA, at least qualitatively. E’, E’’, and tan δ can be divided into the same zones as for PLA, with slight shifts in temperatures and small differences in the magnitude of the responses. From this analysis, the T_g_ related to PLA is estimated as 64 °C, and the onset of T_cc_ is at 86 °C, suggesting that the presence of PHA in PLA decreases the transition temperatures in the blend. The variations shown in the behavior of PLA–PHA may be related to increased chain mobility of amorphous PLA due to the presence of PHA. The DMA response of PHA is quite different to that of the other two filaments investigated. E’ and E’’ of PHA (Figure 11c) start at 2.3 GPa and 165 MPa at 25 °C, respectively. Since this polymer at room temperature is above its T_g_, only a constant drop in E’ occurs until 160 °C, where it decreases dramatically. The drastic decrease in E’ is due to the onset of T_m_ where the material flows, as observed in DSC. Tan δ remains almost constant until 178 °C, where it suddenly increases due to material melting. This is because at this temperature the material is already above its T_m_ (172 °C according to DSC). The fact that PLA has the highest E’ at 25 °C, followed by PLA–PHA and PHA, is consistent with the trends of the elastic moduli reported in Table 5. The numerical difference between the elastic moduli obtained through tensile tests and the storage moduli at room temperature is due to the viscoelastic component in the behavior of the polymers [64].

## 4. Conclusions

The mechanical and dynamic mechanical properties of commercial fused deposition modeling (FDM) filaments made of PLA, PHA, and a PLA–PHA blend (88:12 wt.%) were investigated and correlated with their measured physicochemical properties. The PLA filament is the most amorphous, with a crystallinity of ~2%, while the PHA filament exhibits the highest crystallinity (45–50%). The PHA filament comprises a mixture of PHB and PHBV.

The PLA–PHA blend is partially immiscible; its T_g_, T_cc_, and T_m_ are slightly lower than those of PLA due to the presence of PHA, as observed in DSC and DMA. Additionally, the decomposition temperature of the blend is higher than that of each polymer separately, as the presence of PHA reduces the mobility of PLA chains. PHA thermally degrades ~50 °C earlier than PLA but forms stable compounds from its C=O bond that remain stable at temperatures above 300 °C.

During fused deposition modeling (3D printing), the first bonds to break are C–O bonds, leading to an increased relative presence of C–C bonds in all three polymers, as determined using XPS. In the case of the PHA, during additive manufacturing, the chains rearrange allowing an increase in crystallinity (~5%). In the case of PLA and the blend, crystallinity is maintained after melting (FDM) as it is predominantly amorphous.

PLA renders adequate stiffness and tensile strength to the PLA–PHA blend. The presence of PHA spherulites at low concentration (12 wt.%) generate synergistic effects greatly increasing the elongation at break and toughness of the blend. The inclusion of PHA in the blend increases the PLA’s ultimate strain by 33% and its toughness by 50%, sacrificing mechanical strength by only 6%. This is due to the spherulite morphology of PHA in the continuous PLA phase and likely the formation of hydrogen bonding between both phases.

The dynamic mechanical (DMA) response of the PLA–PHA blend is dominated by that of the PLA, given its much higher concentration (88:12 wt.%). However, the blend shows small shifts in E’, E’’, and tan δ toward lower temperatures, indicating the interaction and increased mobility of polymeric chains in its amorphous part. The information obtained from the analysis of the mechanical and thermal behavior of polymer filaments and the relationship with their physicochemical properties is useful in the continuous improvement process of FDM manufacturing of polymer parts, scaffolds, and structures.

## Figures and Tables

**Table 1:** Parameters obtained from DSC analysis.

Matrix	Heating	T_g_ (°C)	T_cc_ (°C)	PLA	PHA		$X_{c}^{DSC}$ (%) Equation (2)
				T_m1_ (°C)	T_m2_ (°C)	T_m3_ (°C)	
PLA	First	64.6	121.3	148.4	-	-	1.5
	Second	63.5	122.8	148.2	-	-	1.7
PHA	First	-	-	-	-	172.0	47.8
	Second	-	-	-	162.4	171.2	50.1
PLA/PHA	First	61.0	126.0	150.7	-	-	0.3 (PLA)
	Second	60.5	117.0	147.8	-	-	0.6 (PLA)

**Table 2:** Degradation temperatures and residual mass of polymeric filaments.

Matrix.	PHA		PLA		T_fd_ (°C)	Residual Mass at 500 °C (%)
	T_id1_ (°C)	T_dmax1_ (°C)	T_id2_ (°C)	T_dmax2_ (°C)		
PLA	-	-	319.3 ± 1.2	373.7 ± 1.5	390.3 ± 1.5	0.3 ± 0.1
PHA	262 ± 1.7	296.3 ± 5.5	-	-	315.7 ± 5.1	11.5 ± 0.1
PLA–PHA	292.0 ± 1.7	315.0 ± 1.0	331.0 ± 1.7	374.0 ± 1.0	394.7 ± 1.5	0.3 ± 0.1

**Table 3:** Concentration of elements present in the polymeric filaments and FDM samples.

Matrix	Type of Sample	C (%)	O (%)	Ca (%)	Na (%)	Cl (%)	N (%)	C/O
PLA	FIL	83.6	11.7	0.59	1.51	0.78	1.8	7.15
	FDM	89.0	9.1	-	0.81	0.21	0.86	9.76
PHA	FIL	70.2	17.6	1.48	1.03	0.98	2.11	3.99
	FDM	86.9	11.8	-	0.71	0.61	-	7.40
PLA–PHA	FIL	90.3	9.70	-	-	-	-	9.31
	FDM	87.1	10.5	0.78	0.92	0.48	0.3	8.32

**Table 4:** Relative bond concentration obtained from the XPS deconvolution analysis.

Element		C1s			O1s	
Binding		C–C (%)	C–O (%)	C=O (%)	C–O (%)	C=O (%)
PLA	FIL	66.8	29.4	3.70	81.0	19.0
	FDM	77.2	15.5	7.4	93.5	6.5
PHA	FIL	66.4	24.3	9.4	79.5	20.5
	FDM	63.8	29.1	7.2	65.2	34.8
PLA–PHA	FIL	58.6	32.3	9.01	84.4	15.6
	FDM	70.6	25.8	3.53	93.0	6.99

**Table 5:** Tensile properties of polymeric filaments. The last column corresponds to the mixture rule.

Filament	PLA	PHA	PLA–PHA	PLA–PHA Equation (4)
Elastic modulus (GPa)	1.5 ± 0.2	0.5 ± 1.6	1.3 ± 0.3	1.4
Tensile strength (MPa)	50.6 ± 0.3	21.1 ± 3.8	47.4 ± 0.2	46.9
Elongation at break (%)	38.1 ± 3.2	16.2 ± 1.8	51.4 ± 9.8	35.7
Toughness (J/m^3^)	16.1 ± 1.5	1.1 ± 0.2	23.8 ± 4.4	14.2

## Data Availability

The data presented in this study are available upon request from the corresponding author.

## Supplementary Materials

The following supporting information can be downloaded at: https://www.mdpi.com/article/10.3390/polym16081062/s1, Figure S1: Test rig and gripping of filaments with the special jaws for tension testing; Figure S2: High resolution XPS windows of the FIL samples, showing deconvolutions; Figure S3: Specific XPS spectra of FDM samples. Table S1: Analysis of FTIR bands of the polymeric filaments shown in Figure 2; Table S2: Analysis of the Raman bands of the polymeric filaments shown in Figure 3.
