# Supplementary material for: Mechanical, Thermal, and Physicochemical Properties of Filaments of Poly (Lactic Acid), Polyhydroxyalkanoates and Their Blend for Additive Manufacturing
Source: Polymers (Basel). 2024 Apr 11;16(8):1062. doi: 10.3390/polym16081062 (PMC11053644; doi:10.3390/polym16081062)
Supplement: Supplementary file 1 [file polymers-16-01062-s001.zip › polymers-2940798-supplementary.pdf]

Mechanical, thermal and physicochemical properties of filaments of poly (lactic acid), polyhydroxyalkanoates and their blend for additive manufacturing

L. Itzkuautli Mondragón-Herrera[1], R.F. Vargas-Coronado[1], H. Carrillo-Escalante[1], J.V. Cauich-Rodríguez[1], F. Hernández-Sánchez[1], C. Velasco-Santos[2], F. Aviles[1*]

[1] Centro de Investigación Científica de Yucatán, A. C., Unidad de Materiales, Calle 43 No. 130 x 32 y 34, Col. Chuburná de Hidalgo, C. P. 97205 Mérida, Yucatán, Mexico

[2] Tecnológico Nacional de México/Instituto Tecnológico de Querétaro, División de Estudios de Posgrado e Investigación, Av. Tecnológico s/n, esq. Gral. Mariano Escobedo, Col. Centro Histórico, 76000 Santiago de Querétaro, Querétaro, Mexico

* Correspondence: faviles@cicy.mx

Supplementary information

S1. Characterization of filament tensile properties

Uniaxial tensile tests were conducted in a Shimadzu AG-I universal testing machine using special grips for circular filaments, as shown in Figure S1. These specialized grips for fibers/filaments require stretching the filament by looping it at each end and securing it with hydraulic clamps. The test was conducted using a 500 N load cell and a crosshead speed of 20 mm/min.

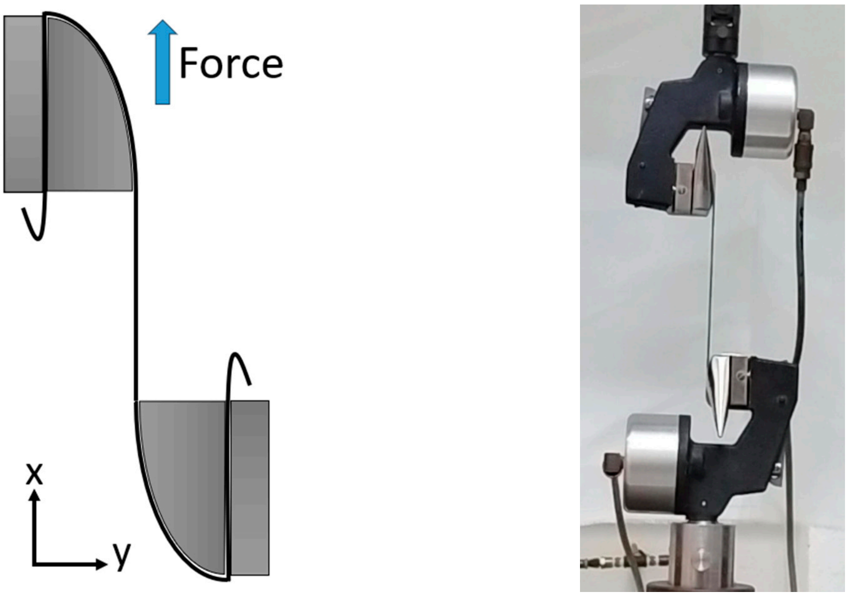

(a) (b)

Figure S1. Test rig and gripping of filaments with the special jaws for tension testing. a) Schematic representation, b) actual photograph.

S2. Physicochemical analysis of polymeric filaments

Table S1 lists the FTIR of each polymer filament discussed in Fig. 2, correlating the wavenumber with the bond type. The discussion of the bands shown in the FTIR is presented in section 3.1.1 of the main paper.

Table S1. Analysis of FTIR bands of the polymeric filaments shown in Fig. 2.

Wavenumber (cm^{-1})			Bond type
PLA	PHA	PLA/PHA	
751	–	752	(α – CH_3)
865	895	867	ν (O – CH – CH_3)
955	–	956	*r* (C – CH_3)
–	973	980	*r* (C – CH_3)
1041	1047	1042	*r* (C – CH_3)
1079	1098	1080	ν_s (C – O – C)
1127	1130	1127	ν (C – O – C)
1179	1176	1180	ν_{as} (C – O – C) / τ (CH_3)
1265	1269	1265	ν_{as} (C – O – C)
1360	1358	1360	δ_s (– CH_3)
1381	1378	1381	δ_s (– CH_3)
1452	1454	1452	δ_{as} (– CH_3)
1745	1721	1745	ν (C = O)
2852	2854	2852	ν_{as} (– CH_3)
2924	2930	2924	ν_s (– CH_3)
2944	–	2944	ν_s (– CH_3)
2994	2977	2994	ν_{as} (– CH_3)
–	3437	–	ν (O – H)

Table S2 lists the bands present in the Raman analysis of the analyzed polymeric filaments, showing the wavenumber location and correlation with the bond type. The discussion of the bands in the Raman analysis is presented in section 3.1.2. of the main paper.

Table S2. Analysis of the Raman bands of the polymeric filaments shown in Fig. 3.

Wavenumber (cm^{-1})			Bond type
PLA	PHA	PLA/PHA	
**-**	433	448	C – CO
**-**	610	613	C – CH_3 / ν (C-CO)
**874**	835	873	ν (C – COO)
**-**	1055	1048	ν (C – CH_3)
**-**	1100	1131	r_{as} (CH_3) / ν_s (C – O – C)
**1298**	1361	1300	δ_s (CH_2)
**1455**	1455	1454	δ_{as} (CH_3)
**1775**	1730	1767	ν (C=O)
**2882**	2877	2883	ν (CH)
**2946**	2933	2946	ν_s (CH_2)
**3001**	2998	3000	ν_{as} (CH_3)

A deconvolution of the XPS carbon and oxygen bands is shown in Figure S2. The deconvoluted spectra revealed C-C, C-O, and C=O bonds in the carbon deconvolution, as well as C-O and C=O bonds in the oxygen deconvolution. The discussion of the deconvolutions in the XPS analysis is presented in section 3.2.4. of the main paper.

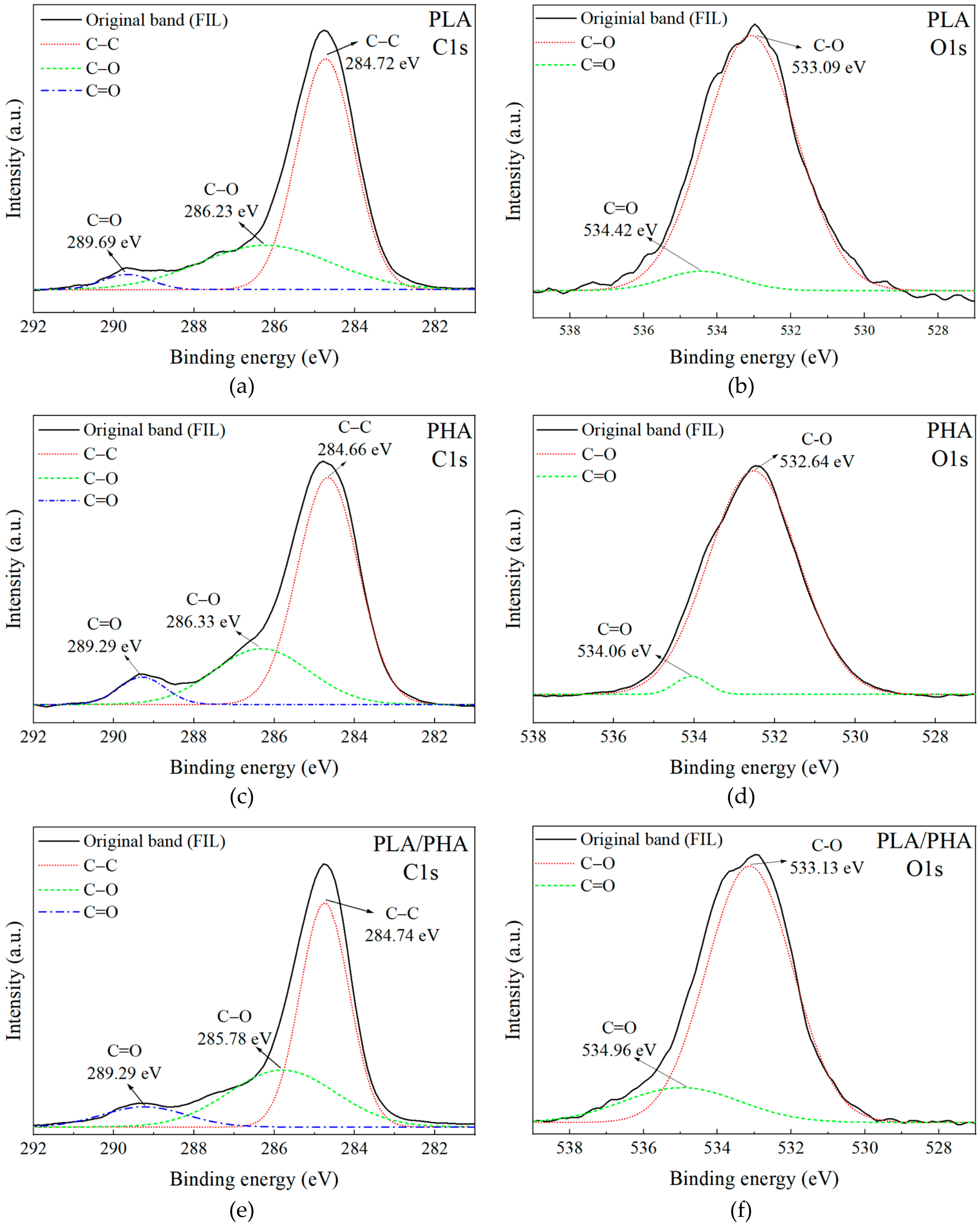

Figure S2. High resolution XPS windows of the FIL samples, showing deconvolutions. (a) C1s of PLA; (b) O1s of PLA; (c) C1s of PHA; (d) O1s of PHA; (e) C1s of PLA/PHA; (f) O1s of PLA/PHA.

S3. Variation of the XPS spectrum with the FDM thermal process

The deconvolutions performed for the high-resolution XPS spectra of the FDM samples are shown in Figure S3. From these deconvolutions, the bonds and variations caused by the thermal process undergone by the FDM filament during additive manufacturing can be analyzed. This is presented in section 3.2.4. of the main paper.

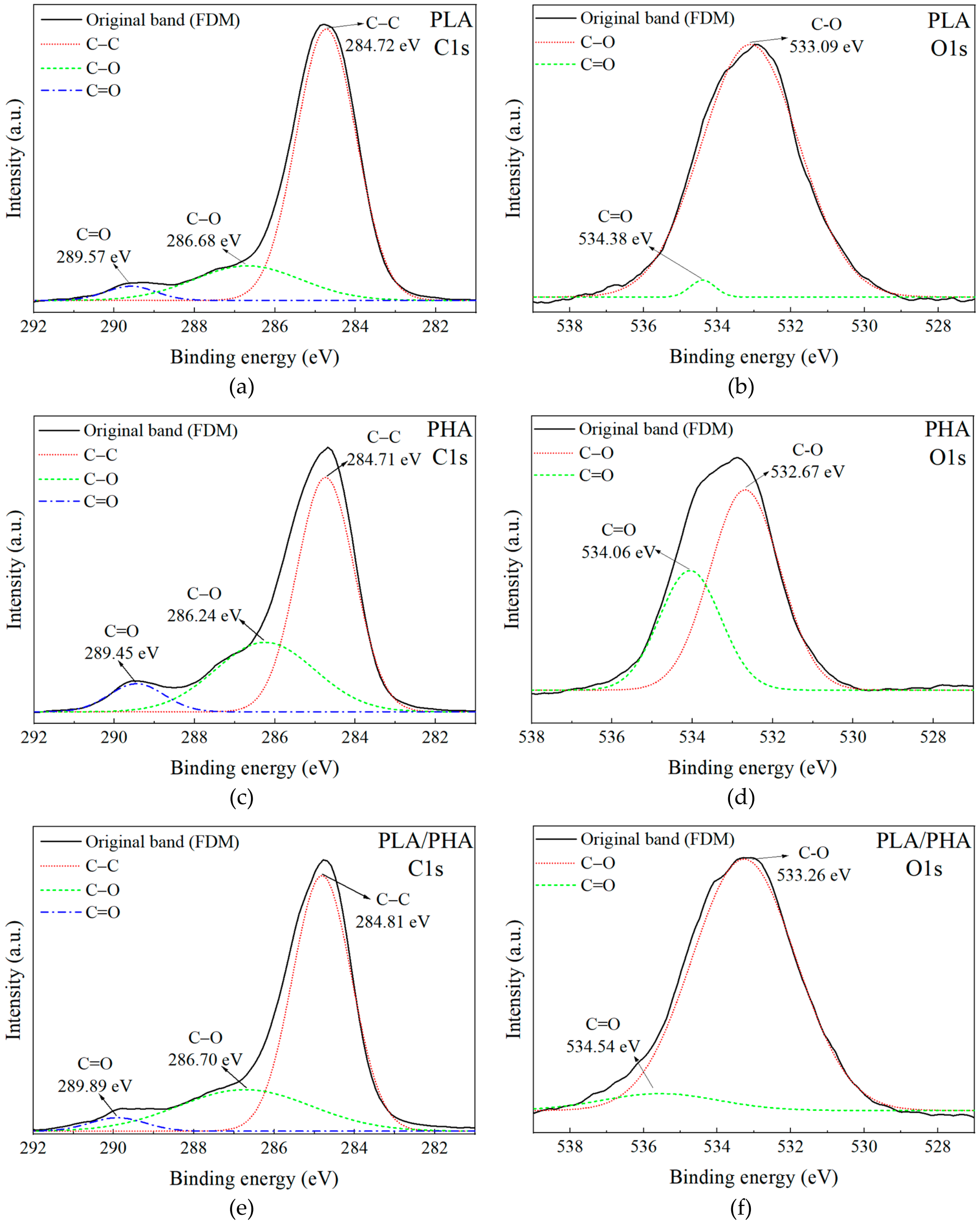

Figure S3. Specific XPS spectra of FDM samples (a) C1s of PLA; (b) O1s of PLA; (c) C1s of PHA; (d) O1s of PHA; (e) C1s of PLA/PHA; (f) O1s of PLA/PHA.
